# Retraction: microRNA526b servers as a prognostic factor and exhibits tumor suppressive property by targeting Sirtuin 7 in hepatocellular carcinoma

**DOI:** 10.18632/oncotarget.28869

**Published:** 2026-04-24

**Authors:** Xin Liu, Liu Yang, Jianfeng Tu, Wenwei Cai, Meiqi Zhang, Zhangxuan Shou, Yingmin Yao, Qiuran Xu

**Affiliations:** ^1^Key Laboratory of Tumor Molecular Diagnosis and Individualized Medicine of Zhejiang Province, Zhejiang Provincial People’s Hospital, People’s Hospital of Hangzhou Medical College, Hangzhou, Zhejiang Province 310014, China; ^2^Department of Emergency, Zhejiang Provincial People’s Hospital, People’s Hospital of Hangzhou Medical College, Hangzhou, Zhejiang Province 310014, China; ^3^Department of Pharmacy, Zhejiang Provincial People’s Hospital, People’s Hospital of Hangzhou Medical College, Hangzhou, Zhejiang Province 310014, China; ^4^Department of Hepatobiliary Surgery, The First Affiliated Hospital of Xi’an Jiaotong University, Xi’an, Shaanxi Province 710061, China; ^*^These authors have contributed equally to this work

**This article has been retracted:** Oncotarget’s Image Forensics investigation of this paper has identified multiple instances of internal duplications and external overlaps with earlier or concurrently published unrelated articles. These include:

Internal Duplications:

- Figure 2C, 2F: Transwell assay images contain overlaps within Figure 2C and between Figures 2C and 2F. These also overlap with images in Figure 6C. The revised Figure 2 provided for correction also contained overlaps in 2C and between 2C and 2F.
- Figure 6C: The Transwell assay NC migration panel overlaps with the invasion panel, and two invasion panels overlap with each other.
- Figure 8: The Western blot for Snail is a duplicate of the blot for Vimentin in Figure 9.

External Duplications:

- Figure 3B: Two panels of lung tissue H&E staining were previously published in Figure 4D of an unrelated 2014 paper [1] and were also found in 2017 and 2018 publications [2, 3].
- Figure 7B, 7D: Bright field microscopy images of hepatocellular carcinoma cells were previously published as epithelial cancer cells in Figure 3A of a 2014 paper [4].
- Figure 8: Western blot images were duplicated in Figures 5 and 7 of [5], Figure 6C of [6], and Figures 4 and 6 of [7].
- Figure 9: Western blot images were reproduced in Figure 5 of [5] and Figure 6C of [6].
- Supplementary Figure 1: The Western blot image was found in Figure 4B of [8].

Additionally, the cell lines SMMC-7721 (used as a hepatocellular carcinoma line) and LO2 (used as a human immortalized normal hepatocyte line) are known to be contaminated with HeLa cervical cancer cells [9, 10].

Although some original data was provided on PubPeer, the Oncotarget Integrity Office has not received evidence verifying its originality. Furthermore, the authors provided a corrected Figure 2, but the corrected figure contained internal overlaps. These issues raise significant concerns regarding the reliability of the data and conclusions. Consequently, the Editorial has been made to retract this paper. Oncotarget has reached out to all authors to confirm this retraction, but received no response.

Original article: Oncotarget. 2017; 8:87737–87749. 87737-87749. https://doi.org/10.18632/oncotarget.21209
